# Rural–urban and socio-demographic differentials in perceived health state among aging population in Ghana

**DOI:** 10.1186/s41043-023-00433-y

**Published:** 2023-09-01

**Authors:** Richard Boateng, Alfred Edwin Yawson, Prince Owusu Adoma

**Affiliations:** 1 University of Education, Winneba, Ghana; 2 University of Ghana, Legon, Ghana

**Keywords:** Aging population, Perceived health state, SAGE 2, Ghana, Vulnerability differentials

## Abstract

**Background:**

The variations in health between rural and urban population have become an increasingly significant public health concern in developing countries including Ghana where urbanization is occurring. Whereas urbanization results in improved access to healthcare services, the concomitant negative consequences of urbanization coupled with unfavorable compositional and contextual attributes can affect the health of populations. The study sought to examine the effect of rural–urban residence and selected socio-demographic factors on perceived health state among aging population by employing a nationally representative data collected by the WHO from 2014 to 2015.

**Methods:**

The data were derived from the WHO Study on Aging wave 2 released in 2019. A total of 4511 individuals, made up of 1018 adults between 18 and 49 years and 3493 respondents within the ages of 50 years and above, were involved in this study. The study examined the Spearman’s rho correlations between perceived health, rural–urban residence, age, sex, marital status, ever schooled, current work state, religion, and regional location. Subsequently, the study employed a multivariable ordinal logistic regression model to test the effect of the selected biosocial and contextual variables on perceived health state.

**Results:**

The selected socio-demographic variables significantly correlated with health state, except for rural–urban residence. However, the predictive ability of rural–urban residence and the socio-demographic variables on perceived health state were strongly ascertained. It was observed that age, sex, rural–urban residence, and current state of work among aging populations were significant predictors of perceived health state as demonstrated by odds ratios and significant p values. The contextual factor of regional location was the most significant variable that increases the perceived health state of respondents in the study.

**Conclusions:**

Continues engagement in work-related activities, an individual’s age within the aging continuum and regional location coupled with its environmental and ecological attributes, may significantly influence the development of positive perception toward health state, which forms a vital constituent of an individual health seeking behavior.

## Introduction

The role of perceived health as a vibrant subjective indicator in the measurement of an individual's self-assessed health embodies an intuitive recognition of the various clinical and non-clinical aspects of health. Studies have shown that though perceived health is a strong measure for predicting healthcare and help-seeking behavior, individuals tend to exhibit varied responses when asked to rate their overall health state today. Scholars have emphasized the critical need for public health action on aging and the inextricable linkages to the health state of an individual [1, 2].

The levels of vulnerabilities associated with aging can be linked to degenerative issues, complexities, and context-specific situations. Existing frameworks on public health actions of healthy aging have focused on the approach of functional abilities and environmental factors [3–5]. However, it has been argued that environmental, context-specific policy and research gaps tend to exacerbate health inequities among aging populations [6, 7]. These dimensions of research actions render the adoption of worldwide gerontology standards on healthcare and social support systems challenging in many local contexts. Nevertheless, for the adoption of general aging-related health and welfare systems in the global setting, there is the need to examine the context and country-specific trajectories of aging and their concomitant vulnerability differentials focusing on the determinants of perceived health state.

The variances in health between rural and urban population have become an increasingly significant public health concern in developing countries including Ghana where urbanization is occurring. Whereas urbanization results in improved access to healthcare services, the concomitant negative consequences of urbanization coupled with unfavorable compositional and contextual attributes can affect the health of populations. There has been tremendous research interest in the study of aging populations in many advanced countries [8–10]. However, it has been revealed that the application of such study recommendations cannot be applied in totality when dealing with developing and less advanced countries due to the contextual variations [11]. The limitations in global generalization and application of recommendations on aging patterns and help-seeking and healthcare-seeking behavior make the examination of country-specific and contextual vulnerability on aging, and health state trajectories *sin quo non*.

The interconnectivity between aging and perceived health state appears to have been prominent in the arena of aging research [12, 13]. The dearth of knowledge has been the role played by the geo-demographic factors in exacerbating levels of perceived health state among the aging populace. Research activities analyzing linkages between aging and an individual's self-appraisal of health should also consider the geo-demographic factors that affect this consequent variable (perceived health) as they can influence the levels of functional assistance sought for and utilization of healthcare services in most developing countries. This study seeks to analyze the geo-demographic determinants of perceived health state associated with aging. The study specifically explores differential effects of rural–urban residence, age, sex, education, marital status, and employment on health among aging individuals within defined age categories with those from 50 years onwards constituting over 77% and further analyzes variations in the probable responses in health state.

## Methods

### Introduction to SAGE 2

The main source of data for this paper was obtained from the WHO Study on Global Ageing and Adult Health (SAGE) Wave 2. These global data were collected to remedy the dearth of knowledge inaccurate and scientific information on the trajectories of aging and health in low and middle-income countries [14]. Thus, conducting longitudinal surveys across six countries involving Ghana, South Africa, China, India, Mexico, and Russia. Longitudinal survey instruments were administered to persons between the ages of 50 years and above. An additional sample of people between the ages of 18 to 49 years was also interviewed for comparison in each country.

The data collection for SAGE Wave 2 took place from 2012 to 2014 among over 39,533 population across the six countries. In Ghana, a total of 4,702 individuals made up of 3,575 respondents who were 50 years and above and 1,160 respondents between 18 and 49 years participated in the study. The data on SAGE Wave 2 and its protocols have been made accessible based on consent to all the user agreements on the WHO SAGE online resources database (https://www.who.int/data/data-collection-tools/study-on-global-ageing-and-adult-health/sage-waves). This paper relies on the availability of SAGE Wave 2 data to explore the vulnerability differentials associated with aging taking into account residential location and other key demographic variables in Ghana.

### Measured items used and analysis

The analysis was centered on existing primary data from the WHO Study on Global Ageing and Adult Health (SAGE) Wave 2. Data from the individual questionnaire were used in this study. Selected components from the 9 sectioned individual questionnaires were used. The constructs used for this study were selected from the demographic, health state, and care, as well as support sections of the individual questionnaires administered in Ghana.

A detailed statistical analysis was conducted using binary and categorical variables. The combination of dual variable types called for the re-categorization of the ages from 50 + , controlling the missing responses for some measured items, and the application of ordinal logistic regression analysis to ascertain the inter-item odds ratio and statistical significance values at less than 0.05 significance level based on 95% confidence interval. The independent variables measured were age, rural–urban residence, gender, employment, marital status, education, religion, and geographical location. The dependent variable, perceived health state, was selected from the health state, care, and functional impairment section of the WHO SAGE Wave 2 individual data questionnaire. The dependent variable was measured on a 5-point Likert scale ranging from very good, good, moderate, bad, and very bad. A multivariable logistic regression analysis was employed to examine the effect of the independent variables and the dependent in three models based on compositional and contextual factors. Further discussions have been done based on existing literature after the analysis of the empirical results. The subsequent sections in this paper present the empirical findings, discussion, and conclusions.

## Results

### Socio-demographic variables

To ascertain the trajectories of rural–urban and socio-demographic variables in perceived health state among the aging population in Ghana, the study explored variables such as rural–urban residence, sex age, marital status, ever schooled, and current working status of 4511 respondents. The data show that 1827 representing 40.5% of the respondents live in urban settlements, whereas 2684 representing 59.5% of respondents reside in rural settlements. The finding on sex shows that 1870 representing 41.5% and 2641 representing 58.5% of the respondents were males and females, respectively. Although the study focused on aging, as a control comparison measure, the respondents between the ages of 18–49 years who constituted 1018(22.6%) were included in the analysis excluding the ages of 0–17 years. Further, respondents between 50 and 59 years were 1269(28.1%) whereas those between 60 and 69 years were 1079 (23.9%). The final age cohort of 70 years and above constituted 1145 (25.4%) of the respondents (Table 1).

**Table 1:** Selected demographic variables

Variable	Measured item	*N* = 4511 (%)
Rural–Urban	Rural	2684 (59.5%)
	Urban	1827 (40.5%)
Sex	Male	1870 (41.5%)
	Female	2641 (58.5%)
Age group	18 to 49	1018 (22.6%)
	50 to 59	1269 (28.1%)
	60 to 69	1079 (23.9%)
	70 and above	1145 (25.4%)
Marital status	Never Married	342 (7.6%)
	Currently Married	2550 (56.5%)
	Cohabiting	59 (1.3%)
	Separated/Divorced	514 (11.4%)
	Widowed	1046 (23.2%)
Ever schooled	Yes	2561 (56.8%)
	No	1950 (43.2%)
Currently working	Yes	3113 (69%)
	No	1398 (31%)
Religion	None	130 (2.9%)
	Buddhism	2 (0.0%)
	Chinese Trad Religion	36 (0.8%)
	Christianity	3287 (72.9%)
	Hinduism	1 (0.0%)
	Islam	849 (18.8%)
	Primal Indigenous	184 (4.1%)
	Other	22 (0.5%)

The categorization of the age cohorts was done in line with their health and social needs, as also the progression in age and the associated linkages with physical activities. The study also considered marital status as a key factor in the subjective measure of health state. It was observed that 342 (7.6%) had never married, 2550 (56.5%) were married at the time of the survey, and 59 (1.3%) were cohabitating. A total of 514 (11.4%) of the respondents were either separated or divorced, whereas 1046 (23.2%) of the respondents were widowed. The study later sought to examine the linkage between marital status and perceived health state after considering the household level support available to partners in unions. The study examined the number of respondents who have had exposure to formal education. It was revealed that 2561 (56.8%) have ever schooled whereas 1950 (43.2%) have never schooled. This exposure to formal school can play a significant role in the access to basic health information, perceived levels of susceptibility, and severity to conditions that can affect the self-construct of a perceived health state. The study summed the demographic analysis by examining the current work state as this could influence the rate of physical activity, livelihood activities, and financial activity to instigate the debilitating effects of diseases and psychosocial stresses.

### Regional distribution of respondents

In an attempt to examine the spatial-demographic patterns and the perceived health state among the aging population in Ghana, the study examined the regional distribution of the respondents. The Ashanti region had the highest number of respondents as demonstrated by 818 (17.3%), whereas Western and Central followed with 593 (12.4%) and 592 (12.5%), respectively. Brong Ahafo had 521 (11.0%), Greater Accra had 492 (10.4%), followed by Northern 471 (9.9%), and Volta with 412 (8.7%). The Eastern region had 365 (7.7%), Upper East had 248 (5.2%), and Upper West 223 (4.7%) (Table 2).

**Table 2:** Regional distribution of respondents

Region	*N* (%)
ASHANTI	818 (17.3%)
BRONG AHAFO	521 (11%)
CENTRAL	592 (12.5%)
EASTERN	365 (7.7%)
GT. ACCRA	492 (10.4%)
NORTHERN	471 (9.9%)
UPPER EAST	248 (5.2%)
UPPER WEST	223 (4.7%)
VOLTA	412 (8.7%)
WESTERN	593 (12.5%)
Total	4735 (100%)

The data on the regional distribution of the respondents are coterminous with the existing trend in the regional population distribution at the time of the survey based on the 2010 population census data in Ghana. It is important to note that the regional inequities in health needs and health delivery resources tend to exacerbate the perceived health state of inhabitants in specific regions.

### Descriptive statistics on perceived health state and Spearman’s rho correlation analysis

**Fig. 1:**
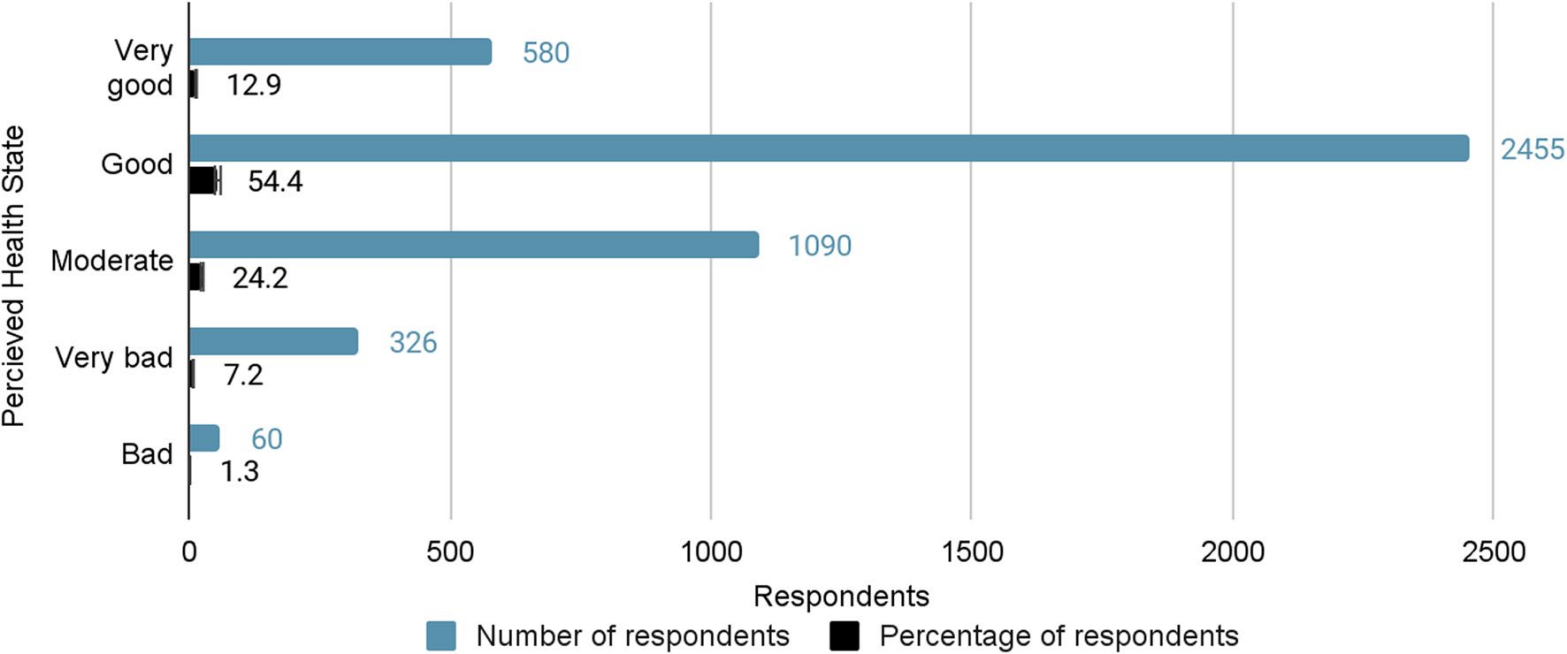
Perceived health state

The descriptive statistics on the perceived health state which is the dependent variable were examined to ascertain the frequency and percentages in the defined categories (Fig. 1).

The respondents who felt their health was bad and very bad constituted 326 (7.2%) and 60 (1.3%), respectively. The highest category very good received relatively lower responses of 580 (12.9%), whereas good and moderate constituted 2455 (54.4%) and 1090 (24.2%) responses, respectively.

Further analysis was conducted to ascertain the inter-item correlations and their statistical significance correction figures in the diagonal parenthesis with double asterisk signify correlation at less than 0.01 significant level and those with single asterisk signify correlation at less than 0.05 significant levels (Table 3).

**Table 3:** Correlation analysis of the measured items

Spearman's rho Correlations		Health state	Age	Sex	Rural–Urban Residence	Currently working	Religion	Ever schooled	Regional Location	Marital status
Health state	Correlation Coefficient	1.000								
Age	Correlation Coefficient	.459^**^	1.000							
Sex	Correlation Coefficient	.077^**^	− .044^**^	1.000						
Rural–Urban Residence	Correlation Coefficient	.042^**^	.067^**^	− .101^**^	1.000					
Currently working	Correlation Coefficient	.294^**^	.317^**^	.034^*^	− .063^**^	1.000				
Religion	Correlation Coefficient	− .013	.008	− .064^**^	.048^**^	.034^*^	1.000			
Ever schooled	Correlation Coefficient	.200^**^	.335^**^	.155^**^	.189^**^	.114^**^	.259^**^	1.000		
Regional Location	Correlation Coefficient	− .020	.029^*^	− .084^**^	.169^**^	.017	.268^**^	.232^**^	1.000	
Marital status	Correlation Coefficient	.301^**^	.415^**^	.312^**^	− .010	.153^**^	− .048^**^	.208^**^	− .044^**^	1.000

^**^Correlation is significant at the 0.01 level (2-tailed)

^*^Correlation is significant at the 0.05 level (2-tailed)

The Spearman rho’s correlation data show that except for religion and regional location which did not show significant correlation values, all the other independent variables significantly correlated with the health state which is the dependent variable at < 0.01 significant levels. Age demonstrated a correlation of 0.459 at the < 0.01 significance level, whereas sex reveal a correlation of 0.077 at the *p* < 0.001 significance level. Rural–urban residence showed a correlation of 0.042 at *p* < 0.001 significance level. The independent variables involving currently working had a positive correlation of 0.294 at *p* < 0.001 significance level whereas marital status and ever schooled depicted correlation values of 0.200 and 0.181 at *p* < 0.001 significance levels, respectively.

### Model fit analysis on the measured items

The study sought to establish an ordinal logistic regression model to examine the effect of a mix of binary and categorical variables on health state which serves as dependent variables in the equation model. The model information exhibits a multinomial probability distribution with a cumulative link function in ascending order. The case processing summary revealed 4511 (100%) population included with no exclusion.

Specifically, the data on categorical variables show the mean and standard deviation of the covariate variables. Religion and location had relatively higher means of 4.88 and 4.72 with standard deviation of 5.89 and 2.91, respectively. Marital status exhibited a mean of 2.83 with a standard deviation of 1.38 whereas the current work state had a relatively lower mean of 1.31 with a standard deviation of 0.46. The goodness of fit indices showed a deviance value/df of 0.67 and chi-square value/df of 1.14.

The omnibus test which compares the fitted model against the threshold-only model shows a significant likelihood ratio chi-square of *p* < 0.001. However, data from the test of model effects showed that only rural/urban residence and religion had relatively lower significant effects as shown by a *p* value of 0.002 and 0003, respectively. The remaining socio-demographic variables measured in the study had statistically significant model effects at a *p* < 0.001 significant level. The viability of the logistic regression model supported the conduct of parameter estimates to Wald chi-square and odds ratio to ascertain the linkages between the antecedent and dependent constructs in the study using 3 models based on existing theoretical constructs.

### Regression coefficient using odds ratio

A multivariable ordinal logistic regression was employed to produce three models based on compositional and contextual factors that influence perception on health state and the data available from the SAGE wave 2 study. The component of model one consists of age, sex, and rural–urban residence as predictors of perceived health state. Model two consist of age, sex, rural–urban residence, employment, religion, and marital status as predictors of perceived health state. Further, model three saw the inclusion of regional location as part of the independent variables in ordinal logistic regression model. The results of the three models analyzed are further presented.

The data reveal the regression coefficients and significance test for each of the covariate variables in the established ordinal regression model. It has been opined that the regression coefficients signify a predicted change in log odds in a higher category on the dependent or consequent variables whiles controlling all other antecedent variables over a unit increase in the antecedent variable under study [15, 16]. Therefore, it is advanced in this study that for a positive estimate, for every one-unit increase on an independent variable, there is a predicted increase as evident in the observed data in the log odds of perceived health state.

This study attempted to place more emphasis on threshold estimates which characterizes the estimates that can be represented as log-odds (odds ratio) leaning toward a particular group or lower margin where scores on other variables are considered to be zero [15] (Table 4).

**Table 4:** Regression coefficient using odds ratio

Variable	Model 1			Model 2			Model 3		
	Exp(B)	95% Wald Confidence Interval for Exp(B) Lower and Upper	*p* value	Exp(B)	95% Wald Confidence Interval for Exp(B) Lower and Upper	*p* value	Exp(B)	95% Wald Confidence Interval for Exp(B) Lower and Upper	*p* value
*Age*									
18 to 49	.051	.042, .061	.000	.098	.079, .123	.000	.102	.082, .128	.000
50 to 59	.211	.180, .248	.000	.324	.270, .388	.000	.336	.280, .404	.000
60 to 69	.390	.333, .458	.000	.517	.436, .612	.000	.525	.443, .623	.000
70 and above	1			1			1		
*Sex*									
Male	.652	.581, .731	.000	.784	.686, .895	.000	.811	.709, .927	.002
Female	1			1			1		
*Residence*									
Urban	.923	.823, 1.036	.173	.926	.819, 1.046	.217	.804	.705, .917	.001
Rural	1			1			1		
*Currently working*									
Yes				.432	.378, .494	.000	.426	.371, .489	.000
No				1			1		
*Religion*									
None				1.776	.729, 4.326	.206	1.844	.750, 4.535	.183
Buddhism				.195	.011, 3.542	.269	.154	.008, 2.840	.208
Chinese trad religion				.958	.334, 2.749	.937	1.375	.459, 4.116	.570
Christianity				.911	.396, 2.094	.827	.864	.373, 2.002	.734
Hinduism				.191	.003, 12.888	.441	.151	.002, 10.514	.383
Islam				.855	.370, 1.975	.713	.861	.372, 1.993	.727
Primal indigenous				.952	.398, 2.278	.912	1.214	.499, 2.950	.669
Other				1			1		
*Ever schooled*									
Yes				.882	.769, 1.012	.073	.784	.680, .905	.001
No				1			1		
*Marital status*									
Never married				.368	.279, 485	.000	.340	.258, .450	.000
Currently married				.712	.605, .838	.000	.713	.605, .839	.000
Cohabiting				.371	.216, .637	.000	.351	.204, .605	.000
Separated/divorced				1.023	.827, 1.266	.836	.997	.805, 1.236	.981
Widowed				1	–	–	1	–	–
*Regional location*									
Ashanti							2.274	1.668, 3.100	.000
Western							1.460	1.060, 2.010	.020
Central							1.238	.899, 1.706	.191
Greater Accra							2.236	1.588, 3.148	.000
Volta							1.220	.869, 1.713	.252
Eastern							2.911	2.058, 4.117	.000
Upper East							.960	.646, 1.425	.839
Brong Ahafo							1.688	1.220, 2.337	.002
Northern							1.383	.993, 1.927	.055
Upper West							1		

*OR* Odd Ratio, *CI* = 95% Wald Confidence Interval for Exp(B), 1 = set to zero because the parameter is redundant

Hence, the study used the regression coefficient and odds ratio to postulate that as scores increase on each of the independent parameters, there is an increased probability of a higher-level perception of perceived health state. In model 1, which was composed of the biosocial factors, it was observed that rural/urban residence was a positive predictor of perceived health as indicated by an odds ratio of 0.923. Moreover, Sex was a significant positive predictor of perceived health state as indicated by an odds ratio of 0.652 with a lower bound Wald interval of 0.581 and upper bound of 0.731 at a *p* < 0.001 significance level. The components of in the age group revealed a positive predictor variable of perceived health state as evident in an odds ratio of 0.390 at a *p* < 0.001 significance level at respondents reaches age 60–69 years. Subsequent analysis was conducted to ascertain the interaction of biosocial and contextual factors on perceived health state.

In model 2, the biosocial and socio-economic factors were analyzed, and it was observed that age group is a predictor variable of perceived health state as evident in an odds ratio of 0.517 at a *p* < 0.001 significance level as respondents progress in age as seen in the 60–69 years group. Moreover, Sex was a significant positive predictor of perceived health state as indicated by an odds ratio of 0.784 with a lower bound Wald interval of 0.686, and upper bound of 0.895 at a *p* < 0.001 significance level. However, in model 2, rural/urban residence was not a significant predictor of perceived health as indicated by an odds ratio of 0.926 with Wald interval of 0.819, and upper bound of 1.046 at a *p* value of 0.217. On the contrary, the socio-demographic variable on currently working was a significant predictor of perceived health state as demonstrated by an odds ratio of 0.432 with a lower bound of 0.378 and upper bound of 0.494 at a *p* < 0.001 statistical significance level. Further, majority of the measured items under marital status with the exception of the element of separation or divorced were significant positive predictors of health state as represented by log odds of 0.368, 0.712, and 0.371 for never married, currently married, and cohabiting, respectively, at *p* < 0.001 significance levels. Although substantial odd ratios were observed under education and religion parameters, these were not statistically significant in the model under review. The variances in the results observed in this model and information obtained from existing literature necessitated the inclusion of geographical location taken into account variances in ecological factors to set up a third multivariable ordinal logistic model to examine the purpose of this study.

In model 3, the biosocial, socio-economic and contextual factors were analyzed. There was a general improvement in the predictive ability of majority of the independent variables in model. It was observed that age group is a predictor variable of perceived health state as evident in an odds ratio of 0.525 at a *p* < 0.001 significance level as respondents progress in age as seen in the 60–69 years group. Moreover, Sex was a significant positive predictor of perceived health state as indicated by an odds ratio of 0.811 with a lower bound Wald interval of 0.705, and upper bound of 0.917 at a *p* < 0.005 significance level. Paradoxically, in model 3, rural/urban residence was a significant predictor of perceived health as indicated by an odds ratio of 0. 804 with Wald interval of 0.705, and upper bound of 0.917 at a p value of 0.001. Further, the socio-demographic variable on currently working was a significant predictor of perceived health state as demonstrated by an odds ratio of 0.426 with a lower bound of 0.371 and upper bound of 0.489 at a *p* < 0.001 statistical significance level. Though the elements under religion had positive odd ratio values, none of these had statistically significant values.

Further, majority of the measured items under marital status except for the element of separation or divorced were significant positive predictors of health state as represented by log odds of 0.340, 0.713, and 0.351 for never married, currently married, and cohabiting, respectively, at *p* < 0.001 significance levels. The rural–urban residence parameter an odd ratio of 0.804 with a lower bound of 0.705 and an upper bound of 0.917 at *p* < 0.005. Similarly, education also has an odd ratio of 0.784 at *p* < 0.005 significance level. Finally, the element of regional location with its ecological variations demonstrated the most significant odd ratios in the model. Specifically, the highly urbanized regions of Ashanti, Greater Accra, and Eastern showed higher odd ratio of 2.274, 2.236, and 2.911, respectively, at *p* < 0.001 statistical significance level.

## Discussion

The study explored the critical roles played by rural/urban residents and selected biosocial, socio-economic, and contextual factors in the perceived health state of individuals within defined aging cohorts. The study acknowledges the fundamental effects of perceived health state in individuals' readiness, cues to actions, and acceptance of positive behavioral outputs and health measures as examined in the health belief model [17]. Central to the issues of a perceived health state are critical issues of perceived susceptibility, severity, and benefits of pursuing recommended primary and secondary health practices. Therefore, the study examined compositional and contextual factors that underpin the perceived health state among the aging population in Ghana using the WHO SAGE Wave 2 data.

Based on the findings, it was ascertained that an individual's age progressively correlates with his/her perception of health state. These findings are coterminous with a previous study which indicated that the potential risk of ill health increase as one progress in age and hence the need to address the health challenges of the people within the aging cohorts [18]. Also, the observance of gender dimensions in the perceived health state call for a thorough examination of gender/sex-related needs among the aging population to influence their perceived health state.

The spearman’s rho correlation analysis further reveals a significant linkage between the current work state and health state as signified by a correlation value of 0.294 at a *p* < 0.001 significance level. The strong relationship also suggests a linkage between individual access to livelihood opportunities leading to reliable income and active engagement in daily activities. These lead to both economic empowerments to meet daily subsistence needs and the conduct of physical activities which enhances bodily maintenance.

It has been asserted that marital status is a vital tool to ascertain the household level resources available for meeting daily social, psychological, and health needs [18]. It is therefore not surprising that a positive relationship was found between marital status and perceived health state.

The study observed a positive relationship between ever schooled (educational status) and health state [19]. An individual's educational status is likely to influence his/her perception of health based on exposure to basic health knowledge and lifestyle management techniques. However, the interplay of selected socio-demographic variables within the study cohort may have accounted for a relatively lower correlation between rural–urban residence and health state. Thus, individual socio-demographic characteristics tend to correlate more with perceived health state.

Subsequently, the predictive ability of individual antecedent variables within the ordinal logistic regression model revealed that the age, sex, rural–urban residence, and current state of work among aging populations were significant predictors of perceived health state as demonstrated by an odds ratios and significant p values in model 3. Further, the observance of age group as an important predictor of perceived health state shown by odds ratio of 0.525 at *p* < 0.001 significance level highlights the potential complementing roles of both favorable work state and age in the development of positive perception toward health state. The study finds it important to stress the progressive engagement of individuals in age and skill-appropriate working activities to help in the development of positive perception toward the health state as this tends to improve national health care utilization and delivery. More significantly, the observance of higher odd ratio among the attributes of regional location with highly urbanized regions dominating demonstrate the significant relationship between regional location and perceived health state. However, the study is limited by reliance on existing data which have a wider scope and diverse objectives.

## Conclusions

Continuous engagement in appropriate and progressive work-related activities and an individual's age within the aging continuum may significantly influence the development of a positive perception of health state while observing the trajectories of other relevant socio-demographic variables within the public health space. The formation of a positive perception of an individual health state can serve as a vital factor in improving care-seeking behavior within the aging community.

## Data Availability

The data are available upon request.
